# U-Th and U-Pb geochronology of Quaternary carbonates

**DOI:** 10.1093/nsr/nwaf078

**Published:** 2025-03-04

**Authors:** Jian Wang, Xiaowen Niu, Le Kang, Hai Cheng

**Affiliations:** Institute of Global Environmental Change, Xi'an Jiaotong University, China; Institute of Global Environmental Change, Xi'an Jiaotong University, China; Institute of Global Environmental Change, Xi'an Jiaotong University, China; Institute of Global Environmental Change, Xi'an Jiaotong University, China

The Quaternary, encompassing the last 2.588 million years (Ma), is pivotal for modern Earth's environment emergence and human evolution, thus holding extensive research interest. Over the past half-century, developments in U-Th and U-Pb radiometric dating methods, tailored to diverse younger and older carbonates in nature, have greatly promoted Quaternary scientific research on multiple fronts in geophysics, paleoclimate, paleoenvironment, paleontology, human evolution, tectonic activities, etc. Here we briefly review recent progress in U-Th and U-Pb dating of Quaternary carbonates, scrutinize challenges encountered, and contemplate future trajectories, with the aim of contributing insights into the ongoing development of U-Th and U-Pb geochronology of Quaternary carbonates.

Viewing U-Th and U-Pb geochronology, optimal precision and sample resolution are largely dependent on the isotope dilution (ID) and *in-situ* analyses facilitated by laser ablation (LA), respectively. Generally, both methods are limited by ionization/transmission efficiency, which is subject to the development of instruments and analytical techniques [1]. A prime example is the high-precision U-Th dating of carbonates using the ID method, where Cheng *et al.* [2] utilized capabilities of multi-collector inductively coupled plasma mass spectrometry (MC-ICP-MS) with an optimized ionization/transmission efficiency of 2%–3%. This allows them to use a set of old speleothems as secular-equilibrium references to attain the most precise ^230^Th and ^234^U half-lives thus far, culminating in the establishment of the world's longest monsoon speleothem record excelled in the ‘x axis’ (absolute time) in ways that are essentially unparalleled in paleoclimate research up to the current U-Th dating limit of ∼640 thousand years (ka) ago [3].

In another forefront, LA U-Th and U-Pb dating techniques for *in-situ* analyses can achieve high spatial resolution up to ∼50 μm. These methods are particularly valuable in resolving the difficulty caused by a limited sample size for dating purposes. For instance, the LA U-Th offers superior stratigraphic clarity compared to solution-based ID approaches for differentiating rock art from dated samples [4]. Moreover, mapping isotopic ratios such as ^230^Th/^232^Th and ^232^Th/^238^U can assess detrital contributions and identify non-closed system behaviors like U loss or recrystallization, thus enabling effective selection of optimal sampling area for dating [4]. A common issue with LA dating lies in the matrix effect due to a lack of ideal reference materials (RMs). Additionally, monitoring analyses of calcite standards with known-ages show significant errors greater than the calibrated values [4], but this may be improved by integrating data from more measurements.

The activity ratio of ^230^Th/^238^U reaches a virtual state of equilibrium after ∼8 half-lives of ^230^Th (∼75 000 years), rendering U-Th dating infeasible at present for ages exceeding ∼600 ka. Alternatively, the U-Pb method, relied on ^238^U-^206^Pb and ^235^U-^207^Pb decay chains (Fig. 1A), was primarily applied to much older samples (>10 Ma) with high U content and relatively low common Pb. Given generally low U contents and less radioactive Pb in Quaternary carbonates, most early studies merely utilized ID approaches to obtain sufficient precision by increasing sample size (∼1 μg of equivalent U). These studies mostly centered on human evolution and fragmented environmental reconstructions [5]. Notably, a cave study based on Italian stalagmites provided an important climate record spanning ∼150 ka in the mid-Pleistocene [6]. It should be emphasized that the U-Pb dating of Quaternary carbonates typically involves the isochron method, so the final uncertainty includes not only analytical errors, uncertainties on the tracers and the decay constants, but also the uncertainty of the isochron slope. While the ID method generally offers higher single-point analytical precision than the LA method, the uncertainty of the slope of the isochron depends not only on the precision of each single-point, but also on the distribution of these points. Normally, the ID U-Pb method is confined to varying degrees by the intricate chemical procedures and sample size, leading to a limited number and range of data points, and therefore nonideal isochrons at times, especially for the Quaternary samples. In contrast, the LA U-Pb method has unique advantages of high sample resolution, small sample size, and easy implementation in preprocess and measurement, normally resulting in more data points and a wider range, facilitating the success rate/better precision of isochrons and leading to its rapid adoption [7,8].

**Figure 1.**
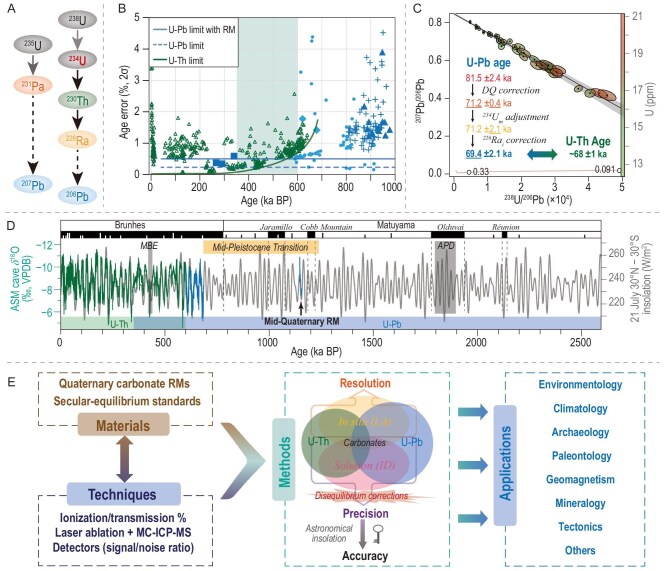
(A) Schematics of ^235^U and ^238^U decay chains for U-Th and U-Pb dating methods. (B) U-Pb (blue) and U-Th (green) age error comparison. U-Th ages are from published Sanbao cave results [3]. The light blue points are LA (dots) and ID (diamonds) dates from XJTU lab [12], while the blue points are published ID dates (squares represent data from ref. [5] and therein, crosses and triangles are obtained through single-aliquot and conventional isochron approaches, respectively [6]). Green and dashed blue lines show minimum age errors derived from existing U-Th and U-Pb dates, respectively. The blue line is the minimum age error of the U-Pb LA method while incorporating the reference material age errors (∼4‰ in [10]). The minimum U-Th error grows exponentially with age, while the minimum U-Pb error remains nearly constant for the last 1 Ma. The shading shows the window suitable for the reciprocal verification between the two dating methods. (C) The youngest speleothem U-Pb corrected age (∼69.4 ka) comparable to the U-Th age within errors. Different U-Pb ages show the effect of DQ (disequilibrium corrections using ^234^U/^238^U_measure_ = 4, and ^231^Pa/^235^U_initial_ = ^230^Th/^238^U_initial_ = ^226^Ra/^238^U_initial_ = 0); ^234^U/^238^U_measure_ error adjustment from 0.004 to 0.2, and ^226^Ra/^238^U_initial_ correction from 0 to 1.6. Underlined value is the specific age from each correction step. The final U-Pb (blue) age is consistent with the U-Th (green) result. (D) Quaternary geological timescale (GTS), July 21 insolation (30°N minus 30°S) (grey) and Asian Monsoon speleothem δ^18^O records (green [3]; blue [12]). The dashed lines indicate geomagnetic reversals, and short white and black lines show geomagnetic excursions. The gray bars are MBE (Mid-Brunches Event) and APD (Atlantic‐Pacific divergence). Yellow bar shows the MPT (Mid-Pleistocene Transition). The Mid-Quaternary RM is the Quaternary calcite standard (SB19-2) from Sanbao Cave, central China [14], and its δ^18^O time series is aligned with the insolation. (E) Schematic diagram showing developments and applications of U-Th and U-Pb carbonate chronologies.

The accuracy of the LA U-Pb age relies intimately on the quality of matrix-matched RMs [9]. Currently used carbonate RMs are too old (>3 Ma) to effectively constrain U-Pb ages for Quaternary carbonates [10]. Of note is that criteria for suitable carbonate RMs differ between U-Th and U-Pb dating methods: while the key U-Th RMs are those presumably in true secular equilibrium, ideal U-Pb RMs for Quaternary samples are practically not. Conventionally, U-Pb RMs are supposed to contain minimal amounts of common Pb. However, Quaternary carbonate RMs with extremely low ^207^Pb may potentially introduce significant uncertainties in ^207^Pb measurement and U-Pb age, as well as having less constraint on the initial Pb isotope ratio. As such, it is acceptable or even favorable for Quaternary RMs to contain a certain amount of common Pb, provided that age precision, reproducibility, and initial Pb measurement are prioritized. Another critical issue regarding Quaternary carbonate U-Pb dating is initial disequilibrium corrections, for isotopes ^234^U, ^231^Pa, ^230^Th, and ^226^Ra (Fig. 1A). For old Quaternary samples, initial ^231^Pa, ^230^Th, and ^226^Ra are generally negligible due to their lower initial contents and shorter half-lives, making ^234^U the main concern. Samples with ^234^U/^238^U activity ratios deviating measurably from 1 (the secular-equilibrium value) can be directly corrected using precisely measured ^234^U/^238^U values [11]. However, ^234^U/^238^U activity ratios analytically indistinguishable from 1 are common in relatively old Quaternary samples, necessitating a reasonable estimation of the initial ^234^U/^238^U or alternatively using ^235^U-^207^Pb dating systematics, despite larger dating errors. Young Quaternary samples with high initial ^234^U/^238^U can achieve remarkable dating precision (e.g. 2σ <3 ka at ∼600 ka [12]) via the high-precision measurement of ^234^U/^238^U using the ID method (Fig. 1B). Additionally, due to the short half-life of ^226^Ra, the potential effect of its initial value is less discussed, but it can be significant particularly for relatively young samples (e.g. <200 ka) or for the U-Pb dates with analytical errors less than 2 ka (Fig. 1C). Advancements in high-precision U-Pb and U-Th dating of carbonates facilitate direct comparison between themselves as well. Noticeably, U-Th dating uncertainties increase nearly exponentially with age, whereas U-Pb dating is not subject to this constraint under ideal conditions, therefore promising higher precision results for samples older than ∼500 ka (Fig. 1B).

The U-Th dating accuracy is established by a thorough agreement of the Asian Monsoon speleothem oxygen isotope (δ^18^O) records over the past 640 ka with the calculated orbital-based insolation curves [2,3]. Due to the nature of the U-Th age equation, systematic offsets result in progressively larger age discrepancies for older samples. For instance, a 0.5‰ offset will cause an age discrepancy of ∼50 years at 50 ka to ∼15–30 ka at 600–650 ka. As the δ^18^O time series broadly follow the insolation without progressive offset, the U-Th dating accuracy is expected to be comparable to insolation calculations or likely within 0.5‰ [2,3]. This has an important implication for the ^14^C calibration, since the older portion (∼25–55 ka) of the current ^14^C calibration (IntCal20) relies heavily on speleothem U-Th ages, and thus has an equivalent accuracy on subcentennial-scales [13]. One might anticipate that the correlation between Asian Monsoon speleothem δ^18^O records and the insolation persists further back in time. If so, the U-Pb dating accuracy and/or standardization could benefit from a similar strategy, leveraging the close correlation between U-Pb dated Asian Monsoon records and the insolation over the older portion of the Quaternary from ∼600 to 2600 ka [12,14] (Fig. 1D).

In general, both U-Th and U-Pb dating precision depends on analytical uncertainties, stemming largely from instrumentations, as well as sample quality and size to varying degrees. Regarding accuracy uncertainties, including those related to the relevant tracers and half-lives, standardizations (or RMs) are essential, since in principle U-Th and U-Pb dating are ultimately a measure of the differential between the measured isotope ratios of samples being dated and the reference standards we use. Therefore, further development of analytical techniques and standardizations is crucial. This includes new mass spectrometer detectors with superior signal-to-noise ratios, enhanced ionization/transmission efficiencies for U, Th and Pb, more effective retarding potential quadrupole (RPQ) for mitigating the tailing effect, improved devices for removing isobaric interferences, refined chemical and instrumental protocols, and the acquisition of a set of better RMs (matrix-matched calcite, aragonite and dolomite) and secular equilibrium standards, etc. These developments would propel carbonate U-Th and U-Pb geochronology to the forefront of Quaternary science and applications (Fig. 1E). Notably, it is anticipated that Asian Monsoon speleothem δ^18^O time series will be further extended back in time [12], providing a series of chronological benchmarks over the entire Quaternary period for exploring the Quaternary environment/tectonic evolutions, landscape changes, climate variations and events, human evolution and civilization history, biodiversity, Earth's magnetic field changes, etc., which in turn accelerates the emergence of speleothem records as the strong fourth leg of the paleoclimate ‘tetrarchate’, complementing marine sediment, ice core and Chinese loess records that are subject to the common difficulty of direct radiometric dating.

## Supplementary Material

nwaf078_Supplemental_File
